# Nutritional Status as a Predictive Biomarker for Immunotherapy Outcomes in Advanced Head and Neck Cancer

**DOI:** 10.3390/cancers13225772

**Published:** 2021-11-18

**Authors:** Meytal Guller, Matthew Herberg, Neha Amin, Hosam Alkhatib, Christopher Maroun, Evan Wu, Hailey Allen, Ying Zheng, Christine Gourin, Peter Vosler, Marietta Tan, Wayne Koch, David Eisele, Tanguy Seiwert, Carole Fakhry, Drew Pardoll, Gangcai Zhu, Rajarsi Mandal

**Affiliations:** 1 Department of Otolaryngology-Head and Neck Surgery, Johns Hopkins University, Baltimore, MD 21287, USA; mguller1@jhmi.edu (M.G.); matthew.herberg@jhmi.edu (M.H.); namin6@jhmi.edu (N.A.); halkhat1@jhmi.edu (H.A.); cmaroun1@jhmi.edu (C.M.); hallen15@jhmi.edu (H.A.); cgourin1@jhmi.edu (C.G.); pvosler1@jhmi.edu (P.V.); marietta.tan@jhmi.edu (M.T.); wkoch@jhmi.edu (W.K.); deisele1@jhmi.edu (D.E.); cfakhry@jhmi.edu (C.F.); 2 Bloomberg–Kimmel Institute for Cancer Immunotherapy at Johns Hopkins, Baltimore, MD 21287, USA; yzheng25@jhmi.edu (Y.Z.); tseiwert@jhmi.edu (T.S.); dpardol1@jhmi.edu (D.P.); 3 Department of Oncology, Johns Hopkins University, Baltimore, MD 21287, USA; ewu17@jhmi.edu

**Keywords:** prognostic nutritional index, body mass index, immunotherapy, head and neck cancer, nutritional status, oncology

## Abstract

**Simple Summary:**

Tumors of head and neck cancer are a heterogenous collection of malignancies affecting the upper aerodigestive tract, the majority of which are head and neck squamous cell carcinomas (HNSCCs). Development of immune checkpoint inhibitors (ICIs) have revolutionized the treatment of advanced HNSCCs, albeit only in a minority of patients. Due to the low clinical response rates for HNSCCs and the potential for immune-related adverse side effects, predictive biomarkers are necessary to characterize patients who are most likely to respond to treatment. Previous studies have indicated that patient nutritional status may impact immune response. However, the effects of pretreatment nutritional status, specifically on immunotherapy-treated HNSCC patients, remains unclear. The aim of our study is to explore the associations between baseline prognostic nutritional index and pretreatment body mass index trends on the outcomes of HNSCC patients treated with anti-PD-1 or anti-CTLA-4 immunotherapy, or both.

**Abstract:**

The association between pretreatment nutritional status and immunotherapy response in patients with advanced head and neck cancer is unclear. We retrospectively analyzed a cohort of 99 patients who underwent treatment with anti-PD-1 or anti-CTLA-4 antibodies (or both) for stage IV HNSCC between 2014 and 2020 at the Johns Hopkins Hospital. Patient demographics and clinical characteristics were retrieved from electronic medical records. Baseline prognostic nutritional index (PNI) scores and pretreatment body mass index (BMI) trends were calculated. Associations between PNI and BMI were correlated with overall survival (OS), progression-free survival (PFS), and immunotherapy response. In univariate analysis, there was a significant correlation between OS and PFS with baseline PNI (OS: HR: 0.464; 95% CI: 0.265–0.814; PFS: *p* = 0.007 and HR: 0.525; 95% CI: 0.341–0.808; *p* = 0.003). Poor OS was also associated with a greater decrease in pretreatment BMI trend (HR: 0.42; 95% CI: 0.229–0.77; *p* = 0.005). In multivariate analysis, baseline PNI but not BMI trend was significantly associated with OS and PFS (OS: log (HR) = −0.79, CI: −1.6, −0.03, *p* = 0.041; PFS: log (HR) = −0.78, CI: −1.4, −0.18, *p* = 0.011). In conclusion, poor pretreatment nutritional status is associated with negative post-immunotherapy outcomes.

## 1. Introduction

Head and neck cancers are a heterogeneous collection of malignancies, typically diagnosed in association with heavy tobacco and alcohol use or infection with the human papillomavirus (HPV) [1]. Squamous cell carcinomas that arise from the mucosal surfaces of the oral cavity, oropharynx, and larynx account for the majority of head and neck cancers [2]. Despite improvements in diagnosis and treatment, prognosis for patients with locally advanced or recurrent or metastatic head and neck squamous cell carcinomas (HNSCCs) remains poor [1,3].

The development of immune checkpoint inhibitors (ICIs), such as anti-programmed cell death protein-1 (anti-PD-1) therapies, have demonstrated promising outcomes and improved overall survival in the treatment of advanced head and neck cancer [4,5]. However, response rates to PD-1 inhibitors in HNSCCs range from only 13% to 20% [5]. Due to the low clinical response rates for HNSCC and the potential for immune-related adverse side effects, predictive biomarkers are necessary to characterize patients who are most likely to respond to treatment. Currently, the most widely used biomarkers are programmed death-ligand 1 (PD-L1) protein expression and tumor mutational burden (TMB). However, recent studies have reported that both biomarkers do not carry a high predictive value [6,7,8]. Although other biomarkers have been evaluated, including HPV status, interferon gamma (IFN-γ) signature, neutrophil to lymphocyte ratio (NLR), and the host microbiome status [9], few studies have investigated nutritional status as a potential biomarker or its effects on immunotherapy outcomes in HNSCC. Patients with head and neck cancer are at a higher risk of malnutrition than the general population, which may be attributed to dysphagia, post-operative complications, alcohol abuse, and cancer-related cachexia [10,11]. Therefore, assessment of nutritional status prior to immunotherapy administration may serve as an additional prognostic tool within the contexts of a patient’s global performance status in predicting treatment outcomes.

The prognostic nutritional index (PNI), calculated by combining serum albumin and total peripheral blood lymphocyte count, is a multiparameter nutritional index developed by Onodera et al. [12]. Although the PNI was originally established to assess the relationship between baseline nutritional status and postoperative complications in cancer patients undergoing gastrointestinal surgery [12], the index was shown to predict outcomes in head and neck cancer patients treated with chemotherapy or radiotherapy, or both [11,13,14,15,16,17]. However, the association between the PNI and immunotherapy outcomes is not well established. A study by Shoji et al. showed that the PNI is significantly associated with immunotherapy response and is an independent prognostic factor for OS and PFS in patients with non-small cell lung carcinoma (NSCLC) [18]. Another study by Peng et al. found that in patients with NSCLC treated with PD-1 inhibitors, a PNI ≥45 is associated with better response, PFS, and OS compared with a PNI <45 [19].

Previous studies also reported an association between body mass index (BMI) and ICI treatment response and survival in melanoma and NSCLC patients, whereby patients with above normal BMI show better clinical outcomes [20,21,22,23]; however, many studies have reported no association between BMI and immunotherapy [24,25,26]. However, because these studies assessed BMI at a single timepoint, it may be hard to distinguish whether baseline nutritional status affected treatment outcomes or whether prior weight loss occurred due to the progression of disease [27]. Interestingly, a recent study by Johannet et al. showed that a low PNI and a decreasing trend in pretreatment BMI rather than baseline BMI category are associated with worse response and survival rates in cancer patients who received immune checkpoint inhibition [28]. This may suggest that longitudinal measurements may more precisely characterize the relationship between BMI and immunotherapy response and survival. The discrepancies between studies on baseline BMI and the lack of reports on pretreatment BMI trend in HNSCC highlight the need for further examination of the prognostic value of BMI in predicting immunotherapy outcomes in head and neck cancer.

Our study aims to investigate the associations between baseline PNI, baseline BMI category, and pretreatment trends in BMI with immunotherapy response and survival in advanced head and neck cancer patients. We conducted a single-center analysis of a cohort of patients with stage IV HNSCC who were treated with anti-PD-1/L1 or anti-CTLA-4 monoclonal antibodies (or both).

## 2. Materials and Methods

### 2.1. Study Design and Patient Population

The study conducted was a retrospective review of 112 patients with stage IV HNSCC who underwent treatment with nivolumab or pembrolizumab between 2014 and 2020 at the Johns Hopkins Hospital. Patient demographics and clinical characteristics were retrieved and included age, sex, smoking and alcohol history, tumor-node-metastasis (TNM) stage, tumor grade, Eastern Cooperative Oncology Group (ECOG) score, HPV status, primary site of cancer, and type of immunotherapy received. To calculate a PNI score, baseline serum albumin and absolute lymphocyte count were collected. To determine the association between pretreatment BMI trends and clinical outcomes, BMI was collected at two timepoints—immediately before immunotherapy infusion (baseline) and long-term at 4–7 months prior (pretreatment). Study exclusion criteria included patients with an ECOG score of 2 and greater and those whose either baseline or pretreatment BMI was <18.5, classified as underweight, as per World Health Organization (WHO) criteria, due to variable health status and low prevalence in the cohort (<2%). Additionally, TMB and PD-L1 tumor proportion score (TPS) were collected for patients who had this information available. This study was approved by the Johns Hopkins University institutional review board.

### 2.2. Evaluation of Baseline PNI Score and Pretreatment BMI Trends

BMI was defined as body weight in kilograms divided by height in meters squared (kg/m^2^) and classified as normal (18.5–24.9), overweight (25–29.9), or obese (≥30), according to WHO guidelines. A pretreatment trend was established by subtracting baseline BMI from pretreatment BMI (pretreatment BMI—baseline BMI), a percentage change was calculated, and patients were categorized into two groups: those whose BMI decreased by ≥2% and those whose BMI decreased by <2% [28]. PNI was calculated using the following equation: 10 × serum albumin level (g/dL) + 0.005 × total lymphocyte count in peripheral blood (cells/μL units) [12]. Patients were divided into two categories, based on established cutoff values: low PNI (<45) and normal PNI (≥45) [19,28].

### 2.3. Statistical Analysis

Statistical analysis was performed using R statistical software (http://www.R-project.org/, version 4.0.1., accessed on 20 June 2020). OS and PFS were calculated from the day of immunotherapy infusion until disease progression or death. Univariate Cox proportional hazards model was used to analyze demographic and clinical characteristics in relation to OS and PFS. Survival curves were generated using the Kaplan–Meier estimator. Patient characteristics across various PNI and BMI distributions in the cohort were analyzed using the Wilcoxon rank sum test for continuous variables and Pearson’s chi-squared test or Fisher’s exact test for qualitative variables. All *p* values ≤0.05 were considered statistically significant.

## 3. Results

Among the study cohort of 112 patients with advanced head and neck cancer who underwent immunotherapy, we identified 99 patients who had albumin and absolute lymphocyte count. After exclusion criteria, there were 99 patients with baseline BMI, 85 of whom had both baseline and pretreatment BMI measurements available. The clinical and demographic characteristics of the cohort are summarized in Table 1.

### 3.1. Analysis of Baseline Characteristics and Survival Outcomes

Of the 99 patients with available baseline serum albumin and total body lymphocytes to calculate a PNI score, 40 (40.4%) had low PNI and 59 (59.6%) had normal PNI. According to WHO guidelines, 49 patients (49.5%) had normal weight (18.5–24.9 kg/m^2^), 34 (34.3%) were overweight (25–29.9 kg/m^2^), and 16 (16.2%) were obese (≥30 kg/m^2^). A total of 42 patients (49.4%) had a pretreatment BMI decrease trend of ≥2% while 43 (50.6%) had a decrease <2%. The relationship between baseline characteristics and OS and PFS is shown in Table 2. Univariate Cox regression analysis showed that better OS was associated with younger age (HR: 0.998; 95% CI: 0.96–0.999; *p* = 0.043), normal baseline PNI group (HR: 0.464; 95% CI: 0.265–0.814; *p* = 0.007), and a decrease <2% in pretreatment BMI (HR: 0.42; 95% CI: 0.229–0.77; *p* = 0.005). Improved PFS was significantly associated with younger age (HR: 0.981; 95% CI: 0.967–0.996; *p* = 0.016) and normal baseline PNI group (HR: 0.525; 95% CI: 0.341–0.808; *p* = 0.003). There were no significant correlations of OS and PFS to the other demographic and clinical characteristics (*p* > 0.05).

**Figure 1:**
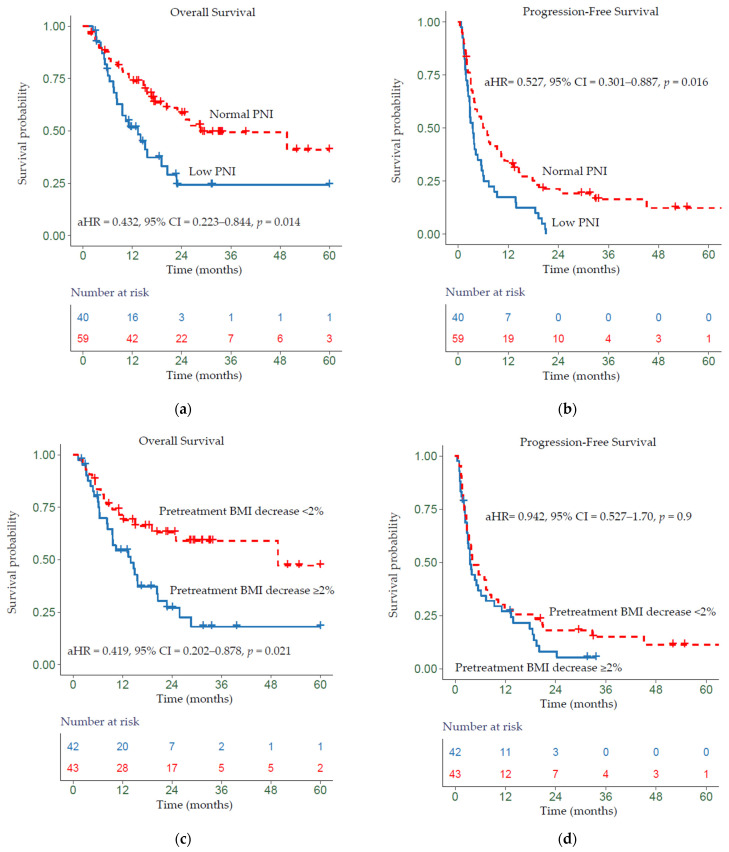
Kaplan–Meier survival curves displaying (**a**,**b**) OS and PFS according to baseline PNI group; (**c**,**d**) OS and PFS by pretreatment BMI trend; aHR—adjusted hazard ratio, calculated from a Cox model controlling for age, gender, HPV status, primary site, TNM, and pretreatment BMI trend or baseline PNI group.

Kaplan–Meier analysis showed that a low PNI was significantly associated with worse OS relative to normal PNI (*p* = 0.014) (Figure 1a) and a worse PFS (*p* = 0.016) (Figure 1b). Patients whose BMI trend decreased ≥2% had a worse OS relative to all other patients (*p* = 0.021) (Figure 1c). Additionally, patients whose pretreatment BMI decreased ≥2% showed slightly worse PFS (non-significant) compared with patients whose BMI decreased by <2% (*p* > 0.05) (Figure 1d). Overall, patients who were obese at baseline showed improved OS (non-significant) and those who were overweight showed better PFS (non-significant) (*p* > 0.05) (see Supplementary Figure S1).

In the multivariate analysis, PNI was an independent prognostic factor for both OS (log (HR) = −0.79, CI: −1.6, −0.03, *p* = 0.041) and PFS (log (HR) = −0.78, CI: −1.4, −0.18, *p* = 0.011). Additionally, age was an independent prognostic factor for PFS (log (HR) = −0.03, CI: −0.06, 0.00, *p* = 0.040) (Table 3). A correlation analysis between age, PNI, and BMI showed that age has minimal–no association with either of these metrics (r = −0.23; r = −0.0086; respectively) (see Supplementary Table S1).

**Figure 2:**
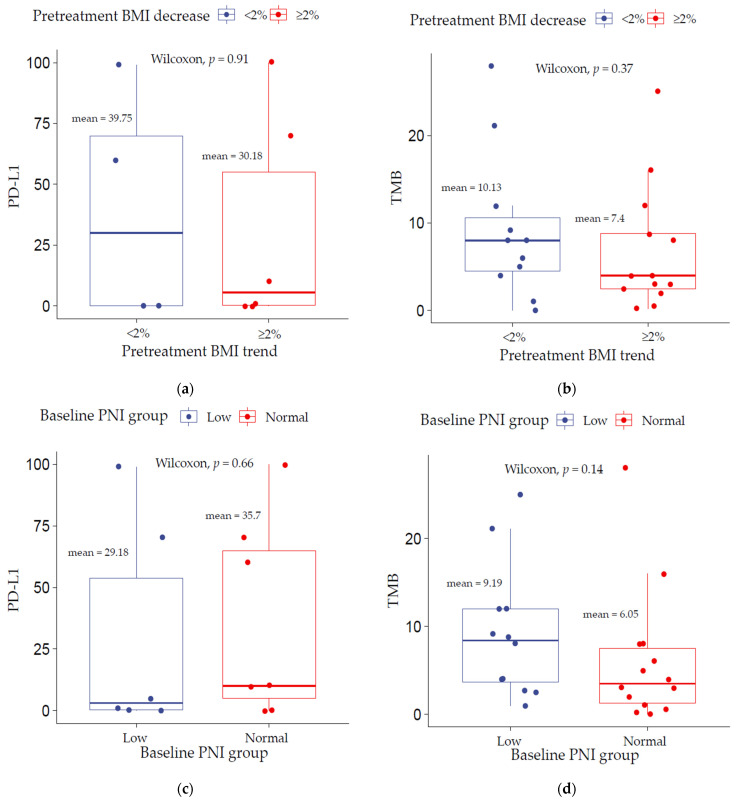
TMB and PD-L1 correlation analysis stratified by (**a**,**b**) pretreatment BMI trend and (**c**,**d**) baseline PNI group.

In total, there were 26 patients with TMB score available and 13 with PD-L1 expression. Of those, 13 patients had both a PD-L1 value and a PNI score and 10 had both a pretreatment BMI trend and PD-L1. Additionally, 25 patients had both TMB and PNI while 24 patients had both TMB and a pretreatment BMI trend. There were no significant differences in PD-L1 and TMB levels between the low decrease (<2%) and high decrease (≥2%) pretreatment BMI trend groups (Figure 2a,b) (*p* > 0.05). Similarly, no statistically significant differences were observed between the low PNI (<45) and normal PNI (≥45) groups (Figure 2c,d) (*p* > 0.05). In the cohort, low TMB but not PD-L1 was found to be significantly associated with decreased overall survival (*p* = 0.014) (see Supplementary Figure S2).

### 3.2. Relationship between PNI, BMI, and Immunotherapy Response

**Figure 3:**
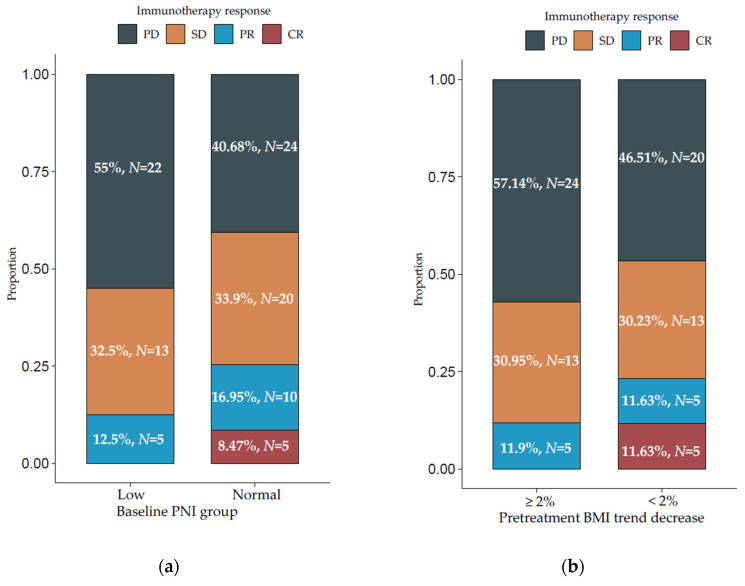
Percentages of immunotherapy responses in patients with (**a**) low vs. normal baseline prognostic nutritional index (PNI) and (**b**) pretreatment BMI trend decrease ≥2% vs. <2%. CR—complete response; PR—partial response; SD—stable disease; PD—progressive disease.

Compared with patients with normal baseline PNI, those with low PNI experienced worse response rates (non-significant) to immune checkpoint blockade. In the normal baseline PNI group, 24 patients (40.68%) had progressive disease (PD), 20 (33.9%) had stable disease (SD), 10 (16.95%) partial response (PR), and 5 (8.47%) complete response (CR). This is compared with 22 (55%) patients with PD, 13 (32.5%) with SD, and 5 (12.5%) with PR in the low PNI group (Figure 3a). There were no complete responders in the low PNI group. Additionally, patients who had a higher decrease in pretreatment BMI trend had worse response rates (non-significant) compared with those with a lower decrease. In the ≥2% decrease in pretreatment BMI group, 24 patients (57.14%) had PD, 13 (30.95%) had SD, and 5 (11.9%) had PR in comparison with 20 (46.51%) with PD, 13 (30.23%) with SD, 5 (11.63%) with PR, and 5 (11.63%) with CR in the <2% decrease group (Figure 3b).

## 4. Discussion

The aim of this study was to investigate the predictive value of nutritional status on immune checkpoint blockade treatment survival and response in patients with advanced HNSCC. To our knowledge, this study is the largest cohort study to investigate the value of baseline PNI, baseline BMI, and pretreatment BMI trend in predicting outcomes to anti-PD-1/L1 or anti-CTLA4 therapies (or both) in head and neck cancer patients. Our findings show that baseline PNI but not baseline BMI category or pretreatment trend are independently associated with treatment survival. Evaluation of baseline PNI showed that patients with low PNI have significantly shorter OS and PFS in both univariate and multivariate analysis. Patients with a pretreatment BMI decrease ≥2% had significantly shorter OS compared with those with a decrease <2% in univariate analysis. While differences in immunotherapy response rates did not reach strong statistical significance, our results showed that patients with a baseline PNI <45 and a pretreatment BMI decrease ≥2% have higher proportion of progressive disease and lack complete response. Overall, these results suggest that poor pretreatment nutritional status associates with worse post-treatment outcomes.

These findings are in line with nutritional status response to conventional therapies, such as chemoradiation [11,13,14,15,16,17]. Nutritional status similarly prognosticates a response to immunotherapy in HNSCC. There are several possible mechanisms that could explain why a poor pretreatment nutritional status could impair immune checkpoint blockade and lead to worse outcomes. Competition for cellular metabolic nutrients (glucose and amino acids) between T cells and tumor cells within the tumor microenvironment can diminish T cells mTOR activity, glycolytic capacity, and cytokine release [29]. This metabolically restrictive environment for tumor infiltrating immune cells, which lack significant energy stores, can further reduce the effectiveness of ICIs [30,31]. Furthermore, changes in adipokines secretion in malnutrition could suppress the immune function and consequently lead to infections and other complications, which further worsen the nutritional status [32]. Alternatively, low nutritional status may be a consequence of aggressive tumor biology rather than being a tumor-independent factor. Therefore, low PNI in patients may serve as a surrogate marker for aggressive disease and would help explain the poor survival outcomes in these patients. However, regardless of the etiology, the association between nutritional status and response to immunotherapy can serve an additional tool in counselling patients regarding their prognosis with immunotherapy treatment.

Our findings are partially consistent with the previous study conducted by Johannet et al., which demonstrated that a low PNI and a decrease in pretreatment BMI trend are independently associated with worse response and survival rates in patients with a variety of solid tumors, including a small number of head and neck cancer patients (*n* = 25) who received treatment with ICIs [28]. However, unlike PNI, we did not find pretreatment BMI trend to be significantly associated with any treatment outcomes on multivariate analysis. Compared with the previous report, our study had a larger head and neck cohort size (*n* = 99) and assessed longer term changes (4–7 months) in pretreatment BMI, as suggested in their limitations section. Furthermore, our study compared baseline PNI and pretreatment BMI trend groups by widely used biomarkers for immunotherapy response and indicated that PNI may be associated with treatment outcomes independent of TMB and PD-L1 expression. This finding suggests the importance of assessing nutritional status as well as TMB and PD-L1 expression before immune checkpoint blockade given to HNSCC patients. Although the number of patients with TMB or PD-L1 in this study was limited, to our knowledge, this is the first investigation on the relationship between PNI, TMB, and PD-L1 status in head and neck cancer.

Although dynamic alterations in BMI fail to be favored as an independent indicator for immunotherapy outcomes in advanced HNSCC in our study, univariate analysis showed that patients with a decrease ≥2% in pretreatment BMI have worse OS compared with those with a decrease <2%. However, no associations between baseline BMI category and survival were observed. Notably, Johannet et al. also did not find significant correlations between baseline BMI category and treatment outcomes. Whether or not patients who are obese and overweight confer an advantage remains controversial in the literature [20,21,22,23,24,25,26]. In fact, the literature on the predictive value of baseline BMI on immunotherapy response and survival show widely inconsistent results [33]. One possible reason for these controversial findings is that weight is a dynamic process and, especially during disease progression, a wider timeframe of serial measurements may reflect a more accurate representation of nutritional status in patients with late-stage disease. Alternatively, the paradox whereby patients with higher BMI have better survival, despite excess weight being a risk factor for developing cancer, may be explained by an increased T-cell dysfunction and expression of PD-1 driven, in part, by leptin signaling, which may be reversed by checkpoint blockade [34]. Interestingly, Kichenadasse et al. and Cortellini et al. reported that elevated BMI is associated with improved survival after immunotherapy, but we should note that the obesity paradox benefit was not reported in the chemotherapy-treated cohorts [22,23]. Therefore, the mechanisms underlying the obesity paradox may not only involve immune cells but also differ by organ of origin [35]. In our cohort, there is no higher expression of PD-L1 in patients with BMI decrease <2% than with ≥2%, which may explain its insignificant role as an independent factor in prognosis. Moreover, although BMI is easily quantifiable, the limitations on BMI may lead to the disputed findings in different studies. For instance, it cannot distinguish skeletal muscle from adipose tissue, which may have different biological effects. Thus, it is increasingly important to assess body composition and sarcopenia as previous studies have suggested that patients with higher muscle content may have better outcomes after treatment with ICIs [25]. Additionally, BMI does not take age or sex into account, which may result in bias, and not always correlate with the metabolism [36]. For instance, muscle mass decreases with age. Therefore, a multitude of factors likely impacts these results, and an elevated BMI alone should not necessarily be viewed as an indicator of better prognosis.

Overall, baseline PNI may provide a promptly available overview of the patient’s nutritional status. We have demonstrated that a low baseline PNI but not BMI significantly impacts immune checkpoint blockade treatment sensitivity in patients with advanced head and neck cancer, who may be particularly prone to malnutrition. There are several limitations to our study. Most notably, the retrospective nature of the study, the limited sample size of head and neck cancer patients who receive immunotherapy, and the lack of a validation cohort. The cohort also mainly consisted of men (87%), who are more likely to develop head and neck cancer compared with women, possibly due to heavier usage of tobacco and alcohol. Thus, future cohort studies with greater numbers of females will be needed to make these findings more generalizable across gender. Another limitation of the study is the question of whether low PNI can be attributed to poor nutritional status or to systematic inflammation, as pro-inflammatory cytokines—such as interleukin-1 (IL-1) and IL-6—regulate production of albumin in hepatocytes [37]. Furthermore, larger cohort studies are necessary to compare the predictive ability of PNI vs. albumin and lymphocytes alone. Additionally, there are other nutrition-related prognostic indices that can be used, such as the gold standard alternatives of the subjective global assessment (SGA) [38] and the patient-generated subjective global assessment (PG-SGA) [39], that may represent a more comprehensive assessment of the nutritional status. Advantages of these tools include the inclusion of dietary changes and symptoms that may adversely affect the nutritional status. Future studies can include these parameters, when data is available, to determine to what extent changes in nutritional status affect response to ICIs. Moreover, future prospective studies are necessary to validate whether managing the pretreatment nutritional status improves treatment outcomes.

## 5. Conclusions

In conclusion, our study suggests a predictive value of pretreatment nutritional status on immunotherapy survival and response in patients with advanced head and neck cancer. Our findings showed that a low baseline PNI is associated with an increased risk for worse post-treatment outcomes. However, we do not have enough evidence to support either baseline BMI category or pretreatment BMI trend as independent prognostic factors in HNSCC patients receiving immunotherapy. Future studies should include a larger sample size and BMI at multiple timepoints. Additionally, prospective studies are necessary to define the role of obesity on immunotherapy outcomes and validate whether managing the pretreatment nutritional status could improve treatment survival and response.

## Figures and Tables

**Table 1:** Clinical and demographic characteristics of the cohort.

Characteristic	N = 99 ^1^
Age	64 (57, 70)
Sex	
Female	13 (13%)
Male	86 (87%)
Smoking Status	
Active	1 (1.0%)
Current	6 (6.1%)
Former	55 (56%)
Never	37 (37%)
Alcohol History	
No	72 (73%)
Yes	22 (22%)
Quit	4 (4.0%)
Unknown	1 (1.0%)
T Stage	
T1–2	42 (42%)
T3–4	40 (40%)
Unknown	17 (17%)
N Stage	
N0–1	24 (24%)
N2–3	57 (58%)
Unknown	18 (18%)
M Stage	
M0	15 (15%)
M1	66 (67%)
Unknown	18 (18%)
Tumor Grade	
Well differentiated	7 (7.1%)
Moderately differentiated	19 (19%)
Poorly differentiated	46 (46%)
Undifferentiated	9 (9.1%)
Unknown	18 (18%)
ECOG Score	
0	4 (4.0%)
1	89 (90%)
2	6 (6.1%)
HPV Status	
Negative	23 (23%)
Positive	39 (39%)
Unknown	37 (37%)
Primary Site	
Non-oropharynx	58 (59%)
Oropharynx	41 (41%)
PNI in baseline	45.8 (42.8, 49.0)
BMI in baseline	25.0 (22.1, 28.5)
BMI pretreatment trend	0.02 (−0.02, 0.07)
IO Name	
Anti-PD-L1	2 (2.0%)
Anti-PD1	88 (89%)
Anti-PD1 and anti-CTLA4	9 (9.1%)
Previous Treatment	
Surgery, chemotherapy, and radiotherapy	44 (44%)
Surgery and radiotherapy	4 (4%)
Chemotherapy and radiotherapy	46 (46%)
Chemotherapy alone	1 (1%)
Radiotherapy alone	2 (2%)
Unknown	2 (2%)

^1^ Median (IQR); *n* (%).

**Table 2:** Univariate cox proportional hazards regression analysis of OS and PFS.

	Overall Survival		Progression-Free Survival	
Characteristic	HR (95% CI)	*p* Value	HR (95% CI)	*p* Value
Age	0.988 (0.96–0.999)	0.043	0.981 (0.967–0.996)	0.016
Sex				
Male vs. Female	1.87 (0.741–4.71)	0.185	1.22 (0.649–2.3)	0.533
Smoking Status				
Current vs. Active	0.59 (0.053–6.56)	0.668	1.42 (0.165–12.2)	0.748
Former vs. Active	1.04 (0.14–7.63)	0.972	1.83 (0.251–13.3)	0.551
Never vs. Active	0.68 (0.09–5.19)	0.714	1.65 (0.224–12.1)	0.624
Alcohol History				
Yes vs. No	0.493 (0.23–1.05)	0.0679	0.734 (0.436–1.24)	0.247
Quit vs. No	1.14 (0.353–3.7)	0.823	0.622 (0.195–1.98)	0.422
T Stage				
T3–4 vs. T1–2	1.26 (0.688–2.32)	0.451	0.911 (0.571–1.46)	0.698
N Stage				
N2–3 vs. N0–1	0.873 (0.435–1.75)	0.703	0.754 (0.454–1.25)	0.274
M Stage				
M1 vs. M0	2.2 (0.856–5.68)	0.101	1.65 (0.837–3.24)	0.148
Tumor Grade				
Moderately vs. Well	0.66 (0.203–2.15)	0.491	0.68 (0.263–1.76)	0.427
Poorly vs. Well	0.721 (0.248–2.09)	0.547	0.632 (0.267–1.49)	0.296
Undifferentiated vs. Well	0.646 (0.161–2.59)	0.537	0.722 (0.249–2.09)	0.549
ECOG Score				
1 vs. 0	1.01 (0.456–2.24)	0.982	1.34 (0.765–2.35)	0.305
HPV Status				
Positive vs. Negative	1.29 (0.613–2.71)	0.504	0.867 (0.501–1.5)	0.61
Primary Site				
Oropharynx vs. non-Oropharynx	1.23 (0.709–2.13)	0.464	1.02 (0.667–1.55)	0.937
IO Name				
Anti-PD-L1 vs. anti-PD1	3.72 × 10^−8^ (0-Inf)	0.996	0.206 (0.0285–1.5)	0.118
Anti-PD1 and anti-CTLA4 vs. anti-PD1	0.801 (0.317–2.02)	0.637	0.952 (0.458–1.98)	0.894
PNI Baseline Group				
Normal (≥45) vs. Low (<45)	0.464 (0.265–0.814)	0.007	0.525 (0.341–0.808)	0.003
BMI Baseline Group				
Obese (*n* = 16) vs. normal (*n* = 49)	0.463 (0.189–1.13)	0.091000	1.08 (0.598–1.94)	0.805000
Overweight (*n* = 34) vs. normal (*n* = 49)	0.793 (0.438–1.44)	0.445000	0.709 (0.441–1.14)	0.157000
BMI Pretreatment Trend Group				
Decrease <2% vs. Decrease ≥2%	0.42 (0.229–0.77)	0.005	0.75 (0.474–1.19)	0.221

**Table 3:** Multivariate analysis of OS and PFS.

	Overall Survival			Progression-Free Survival		
Characteristic	Log (HR) ^1^	95% CI ^1^	*p* Value	Log (HR) ^1^	95% CI ^1^	*p* Value
Age	−0.02	−0.06, 0.01	0.2	−0.03	−0.06, 0.00	0.040
Sex						
Female	-	-		-	-	
Male	0.15	−1.0, 1.3	0.8	0.40	−0.40, 1.2	0.3
T Stage						
T1–2	-	-		-	-	
T3–4	0.32	−0.42, 1.0	0.4	0.27	−0.30, 0.83	0.4
Unknown						
N Stage						
N0–1	-	-		-	-	
N2–3	−0.28	−1.1, 0.58	0.5	−0.31	−1.0, 0.34	0.4
Unknown	−0.16	−1.7, 1.4	0.8	0.23	−1.2, 1.7	0.7
HPV Status						
Negative	-	-		-	-	
Positive	−0.30	−1.8, 1.2	0.7	−0.41	−1.5, 0.65	0.4
Unknown	−0.03	−1.2, 1.2	>0.9	−0.71	−1.7, 0.26	0.2
Primary Site						
Non-oropharyngeal	-	-		-	-	
Oropharyngeal	0.80	−0.49, 2.1	0.2	0.08	−0.87, 1.0	0.9
PNI Group						
Low	-	-		-	-	
Normal	−0.79	−1.6, −0.03	0.041	−0.78	−1.4, −0.18	0.011
Pretreatment BMI Trend						
Decrease ≥2%	-	-		-	-	
Decrease <2%	−0.50	−1.3, 0.34	0.2	0.36	−0.35, 1.1	0.3

^1^ HR—hazard ratio; CI—confidence interval.

## Data Availability

Data is available on request from the corresponding authors.

## Supplementary Materials

The following are available online at https://www.mdpi.com/article/10.3390/cancers13225772/s1, Table S1: Correlation between age, baseline PNI, and pretreatment BMI trend, Figure S1: Kaplan–Meier survival curve displaying OS and PFS by baseline BMI category, Figure S2: Kaplan–Meier survival curves displaying OS according to TMB and PD-L1 group.
