# Supplementary material for: Nutritional Status as a Predictive Biomarker for Immunotherapy Outcomes in Advanced Head and Neck Cancer
Source: Cancers (Basel). 2021 Nov 18;13(22):5772. doi: 10.3390/cancers13225772 (PMC8616447; doi:10.3390/cancers13225772)
Supplement: Supplementary file 1 [file cancers-13-05772-s001.zip › Supplementary Materials_Revised.pdf]

cancers

MDPI

Supplementary Materials

Nutritional Status as a Predictive Biomarker for Immunotherapy Outcomes in Advanced Head and Neck Cancer

Table S1. Correlation between age, baseline PNI, and pretreatment BMI trend

**Characteristic**	**Pearson's r**	***p* value**
Age vs. baseline PNI	-0.0086	0.93
Age vs. pretreatment BMI trend	-0.23	**0.05**
Baseline PNI vs. pretreatment BMI trend	-0.15	0.19

Cancers **2021**, *13*, 5772. https://doi.org/10.3390/cancers13225772 www.mdpi.com/journal/cancers

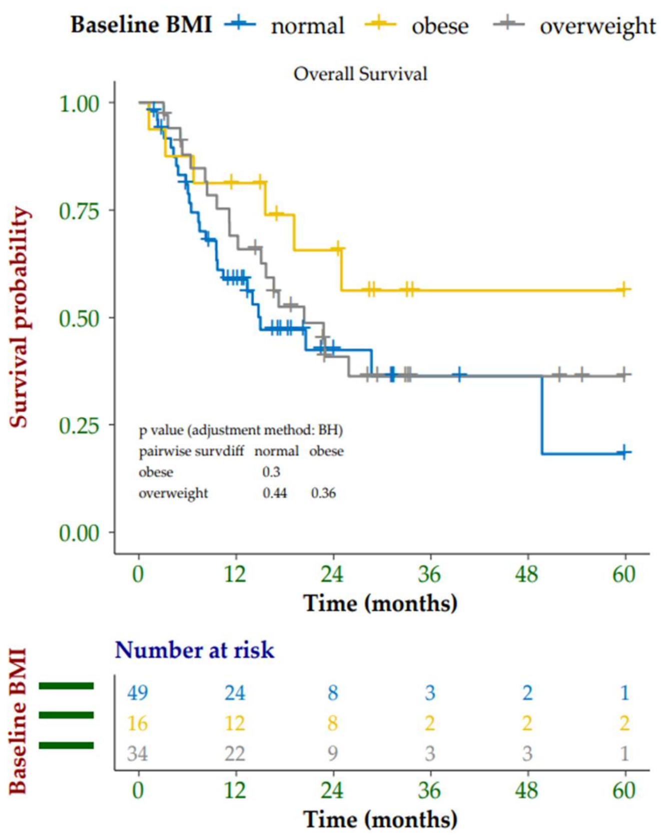

(**a**)

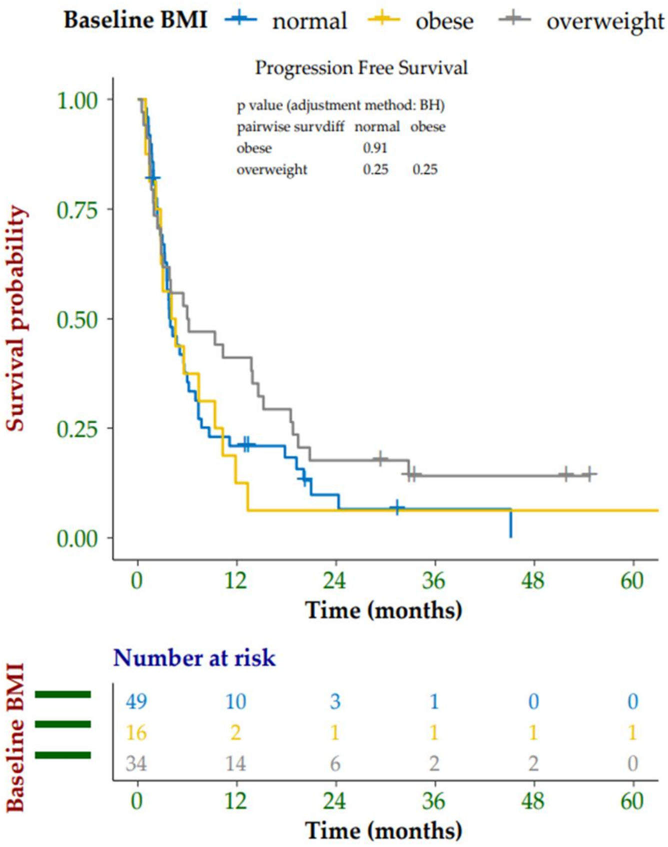

(**b**)

Figure S1. Kaplan-Meier survival curves displaying (a) OS according to baseline BMI group; (b) PFS by baseline BMI group

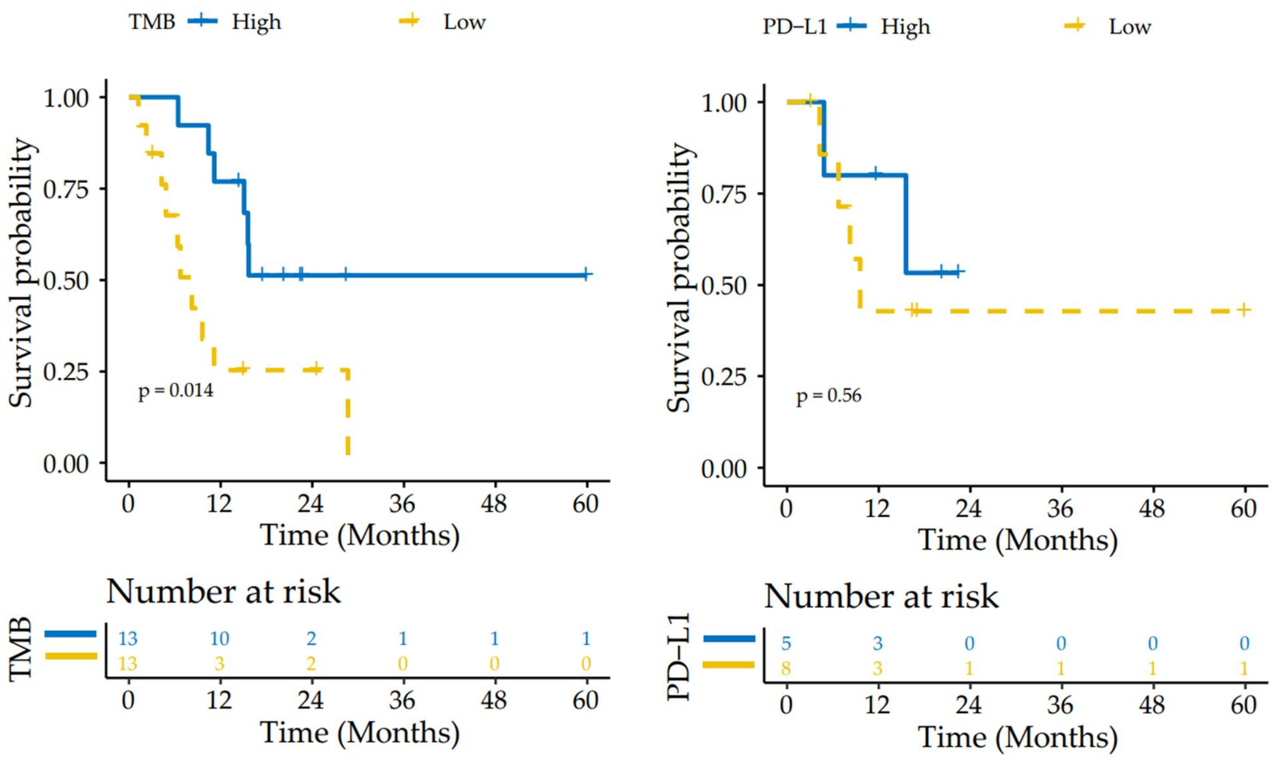

(**a**) (**b**)

Figure S2. Kaplan-Meier survival curves displaying (a) OS according to TMB group; (b) OS according to PD-L1 group
